# Functional status and its related factors among stroke survivors in rehabilitation departments of hospitals in Shenzhen, China: a cross-sectional study

**DOI:** 10.1186/s12883-022-02696-0

**Published:** 2022-05-11

**Authors:** Jing Zhou, Fang Liu, Mingchao Zhou, Jianjun Long, Fubing Zha, Miaoling Chen, Jiehui Li, Qingqing Yang, Zeyu Zhang, Yulong Wang

**Affiliations:** 1 Department of Rehabilitation, the First Affiliated Hospital of Shenzhen University/Shenzhen Second People’s Hospital, Guangdong Province, 3002 Sungang West Road, Futian District, Shenzhen, 518035 China; 2 Shandong University of Traditional Chinese Medicine, Shandong Province, 4655 Daxue Road, Changqing District, Jinan, 250355 China

**Keywords:** Activities of daily living, Longshi Scale, Functional Status, China, Stroke

## Abstract

**Background:**

Many stroke survivors have multiple chronic diseases and complications coupled with various other factors which may affect their functional status. We aimed to investigate the factors associated with poor functional status in hospitalized patients with stroke in Shenzhen, China.

**Methods:**

In this cross-sectional study, four urban hospitals were selected using convenient sampling, and all stroke patients in these four hospitals were included using cluster sampling. The functional status of stroke survivors was evaluated using Longshi Scale. Explanatory variables (factors affecting functional status comprising age, sex, body mass index, smoking, alcohol consumption, complications, and chronic conditions) were collected. Ordinal logistic regression was used to examine which factors were associated with poor functional status.

**Results:**

Stroke survivors with poor functional status accounted for 72.14% and were categorised as the bedridden group based on Longshi scale, 21.67% of patients with moderate functional limitation were categorised as the domestic group, and 6.19% of the patients with mild functional restriction were categorised as the community group. The highest dependence scores were noted for feeding (73.39%), bowel and bladder management (69.74%) and entertainment (69.53%) among the bedridden group, and housework (74.29%) among the domestic group. In the adjusted model, the odds of poor functional status were higher among stroke patients with older age (odds ratio [OR] = 2.39, 95% CI: 1.55–3.80), female sex (OR = 1.73, 95% CI: 1.08–2.77), duration of stroke more than 12 months (OR = 1.94, 95% CI: 1.28–2.95), with pulmonary infection (OR = 10.91, 95% CI: 5.81–20.50), and with deep venous thrombosis (OR = 3.00, 95% CI: 1.28–7.04).

**Conclusions:**

Older adults (age ≥ 60 years) and women were more likely to exhibit poor functional status post-stroke. Pulmonary infection and deep venous thrombosis were related to an increased risk of being dependent on activities of daily living. Therefore, clinical and rehabilitation interventions aimed at preventing or treating these common complications should be addressed to deal with subsequent dysfunction post-stroke. Since all data were obtained in metropolitan areas where the economy is well developed, future studies should be conducted in rural areas and economically less developed cities.

**Supplementary Information:**

The online version contains supplementary material available at 10.1186/s12883-022-02696-0.

## Background

Over the past century, the incidence of stroke has increased dramatically, and stroke has become the third leading cause of physical disability worldwide [1]. It has been estimated that 37–45% of stroke survivors suffer from functional disabilities [2, 3] and that these individuals are dependent on others for one or more activities of daily living (ADLs) [1]. This dependence on others has become one of the most significant factors contributing to a long-term costs for stroke patients [4]. Thus, there is an urgent need to address the disability and associated complications caused by stroke and to improve these individuals’ functional ability and level of independence [1, 5, 6].

Several factors can affect stroke survivors' functional ability. Demographic factors such as age, sex, and body mass index (BMI), as well as lifestyle behaviors (e.g., smoking or drinking), play an important role in the development of functional outcomes of patients with stroke [1, 7–11]. Age influences the long-term functional recovery of stroke patients. Their functional ability at age ≥ 70 decreases gradually between 6 to 30 months after stroke onset, while those aged < 70 have no significant change [10]. The total number of female stroke survivors is higher and their functional outcomes are worse than their male counterparts [8]. Smoking and alcohol consumption are also detrimental to stroke survivors’ functional outcomes [9, 11]. Compared to non-smokers, current and recent smokers tend to have an increased risk of poor functional outcomes at 3 months after acute ischemic stroke [9].

Some complications are also considered essential factors that affect the rehabilitation outcomes of stroke survivors [12–14]. Pulmonary infection, deep vein thrombosis (DVT), and urinary tract infection are the common complications after stroke [1, 14, 15]. Previous studies have shown that the incidence of pulmonary infection can be as high as 33% post-stroke, and that of urinary tract infection can range from 2 to 27% [13–15]. Moreover, these complications influence one another, and the presence of one of these complications increases the likelihood of other complications. In addition, pulmonary infection is an independent risk factor associated with DVT in hospitalized patients with stroke [12]. Even though functional outcome studies among stroke survivors, in general, have shown that the strongest predictor of functional recovery is the initial severity of the stroke [16, 17], few studies have detailed the clinical characteristics of hospitalized patients with stroke [17], especially in rehabilitation departments of urban hospitals in China, as approximately 1115 cases per 100 000 people has experienced a stroke in their lifetime [18].

With the increasing prevalence of multiple chronic conditions (MCCs) in stroke survivors, MCCs have a modest association with post-stroke functional outcomes and contribute considerably to functional impairment [19–23]. In Singapore, a recent study confirmed the relationship between MCCs and post-stroke readmission [24]. A previous study published by our team also found that increased MCCs lead to poorerADL ability in older stroke survivors, although there were no data available on young stroke survivors [21]. Therefore, due to the differences in individual characteristics, studies focused on the association between MCCs and hospitalized stroke survivors are required in China’s clinical settings.

Several studies have explored individual factors such as demographic characteristics, stroke complications, and the presence of MCCs in patients post-stroke and have shown that most of these factors can be controlled through appropriate clinical and rehabilitation interventions in public health. However, there has been no comprehensive research to analyze those factors. It is important to note that a full understanding of the situation of stroke survivors is essential for planning future healthcare resources and identifying appropriate treatment strategies [17]. Hence, comprehensive research on poor functional status may help physicians identify these factors and provide appropriate interventional care as soon as possible.

## Methods

### Study design and setting

The cross-sectional survey was conducted in the rehabilitation department of four hospitals: two secondary hospitals and two tertiary hospitals. These four hospitals were selected from 54 urban hospitals (17 secondary and 37 tertiary) which are equipped with a rehabilitation department in Shenzhen, China, through convenience sampling, as our rehabilitation department collaborates closely with these organizations. Initially, this study aimed to investigate the care needs of inpatients in rehabilitation hospitals. The study protocol was registered in the China Clinical Trial Registration Center (ChiCTR -2000034067) and approved by the ethics committee of the Shenzhen Second People’s Hospital.

### Study participants

**Fig. 1:**
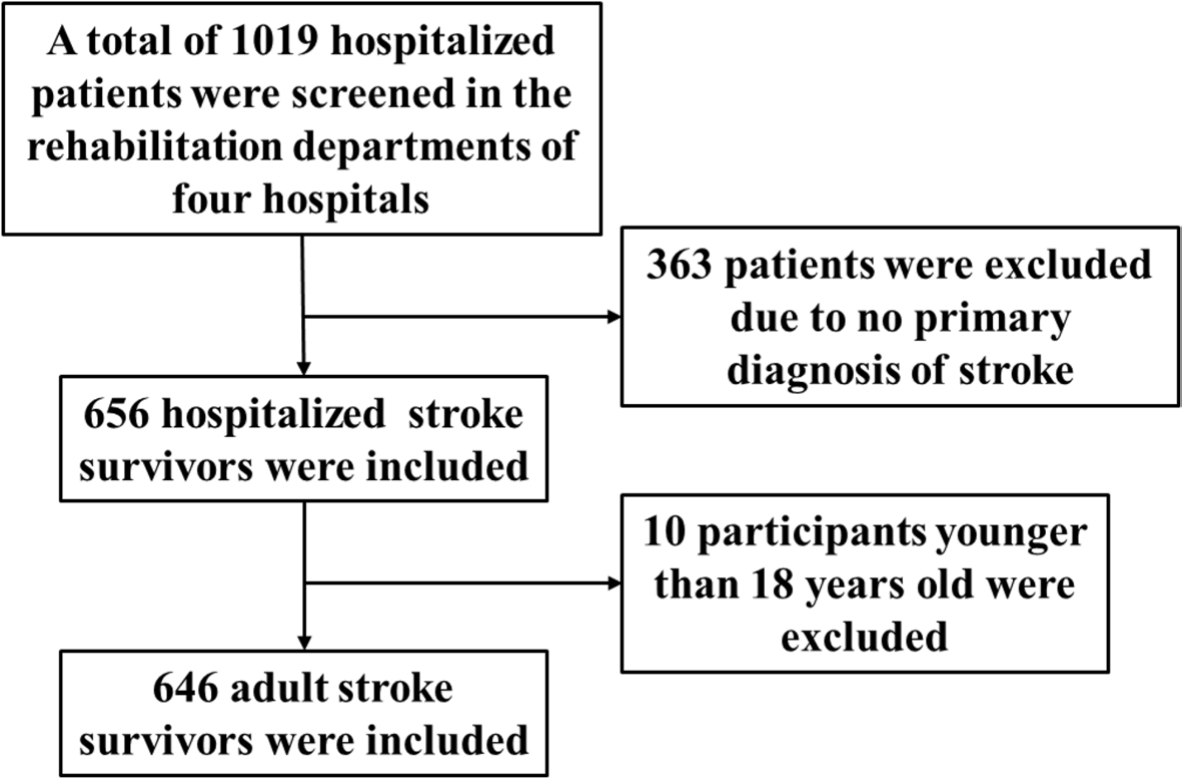
Flow diagram of selection of study subjects

A total of 1019 rehabilitation inpatients in the four hospitals were screened, and all stroke survivors (646) who fit the inclusion and exclusion criteria were recruited using cluster sampling between August 4 and August 22, 2021 (Fig. 1). Among them, 290 (44.89%) and 356 (55.11%) patients were enrolled from two secondary hospitals and two tertiary hospitals respectively. The inclusion criteria were: (1) all patients diagnosed with stroke according to the 10th edition of the International Classification of Disease in the selected hospitals [25]; (2) age ≥ 18 years. The exclusion criteria were: (1) diagnosis of subarachnoid hemorrhage; (2) patients’ inability to give informed consent. The type of stroke was determined based on the initial diagnosis from the patients’ medical history. Before the recruitment, the investigator explained the study contents in detail to the participants. All participants or their caregivers provided written informed consent prior to enrolling in this study.

### Study variables

#### Dependent variable

Most studies of stroke use the Modified Rankin Scale (mRS) to distinguish patients’ functional outcomes. However, mRS does not measure ADL-related functions and is insensitive to subtle differences in functional status between patients [26]. The Longshi scale (LS), the national standard for the daily self-care ability assessment in China, is a visual-based scale that evaluates patients’ abilities based on activity range and dependency [27–29]. Using the Barthel index as a reference, LS can identify the disability of stroke survivors better than mRS [30]. In addition, previous studies have demonstrated that the LS is reliable and valid for disability assessment [27–29]. Thus, in our study, ADL was measured by LS to define functional status as the dependent variable. The participants/their family members or caregivers reported the actual functional status and ADL manifestations of the patients with stroke, including participants with cognitive impairment [30].

**Fig. 2:**
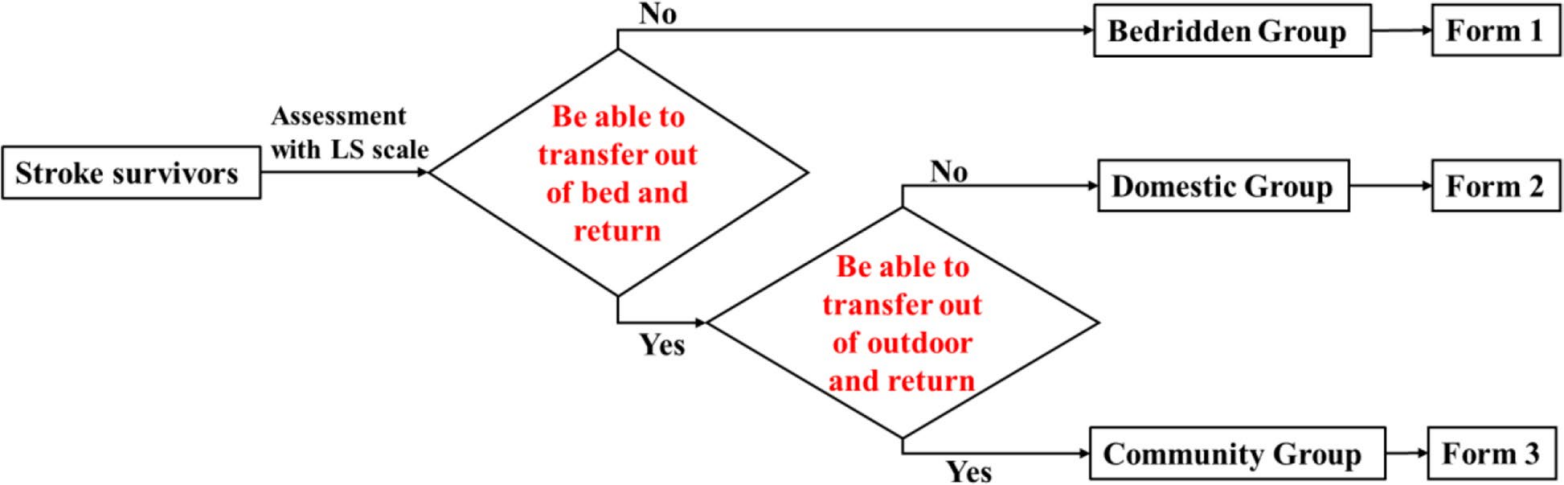
Flow chart of assessment using Longshi Scale

**Fig. 3:**
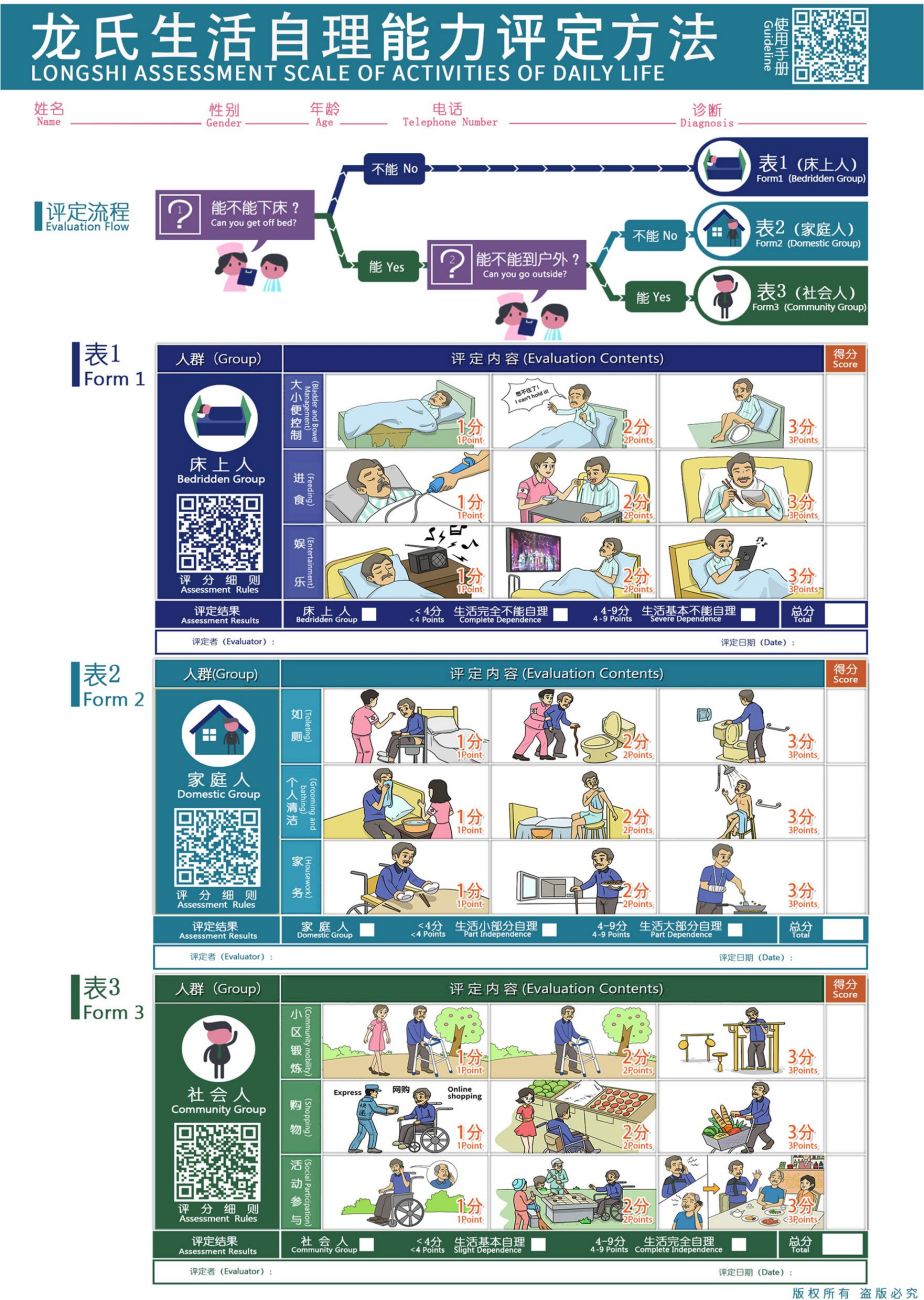
Longshi Assessment Scale of Activities of Daily Life

LS scale assesses participants’ independence or dependence via responses to two questions and assessments of nine activities. The activity levels were divided into three groups (bedridden, domestic, and community groups) depending on the activity range of the participants (Fig. 2) [30]. The participants and/or their family members or caregivers were asked “Can you/he/she get off the bed independently? And can you/he/she go outdoor independently?”, with only two answer options ( “yes” or “no”) available [28]. The bedridden group represents those who cannot get off the bed independently. The domestic group can get off the bed independently but cannot go outdoor independently. The subjects in the community group can go outdoor independently [28]. Next, nine activities (bowel and bladder control, feeding, entertainment, toileting, grooming, housework, community mobility, shopping and social participation) were evaluated to determine the patients’ dependency. In each activity range category, subjects were evaluated on three items (Fig. 3).

Notably, the LS indicates participants’ level of functional status. The community group was considered to have a mild functional limitation (i.e., having a mild problem performing everyday tasks), the domestic group indicates moderate functional limitation (i.e., having a moderate problem performing everyday tasks), and the bedridden group has poor functional status (i.e., having a severe problem performing everyday tasks).

#### Independent variables

In this study, we collected as independent variables data on patients’ age, sex, BMI, smoking history, alcohol consumption history, common stroke complications, and multiple chronic conditions that could be a risk factor and potentially affect the management of stroke survivors. Demographic data were obtained by online questionnaires from participants and their families. Questions about smoking and drinking history were assessed as dichotomous (yes/no) responses. No smoking was defined as those who never smoked in their lifetime, and patients who previously smoked but did not smoke at the time of stroke or those who currently smoke were defined as smokers. Alcohol consumption was defined as habitual consumption of alcoholic beverages before stroke onset [9, 31]. Our study considered three common complications: (1) pulmonary infection, (2) urinary tract infection, and(3) DVT, as well as nine common chronic diseases: (1) hypertension, (2) cardiovascular disease, (3) diabetes, (4) hyperlipidemia, (5) hyperuricemia, (6) chronic obstructive pulmonary disease, (7) chronic renal insufficiency, (8) abnormal liver function, (9) chronic bronchitis.

Data on chronic diseases were collected from patients’ medical and medication histories. This history was examined by trained research assistants, and when there was a diagnosis and treatment of a chronic disease or a chronic disease-related medication was prescribed, it was recorded as “yes” to chronic disease. In addition, patients’ vital signs, including blood pressure, heart rate, blood glucose level, temperature, and oxygen saturation, were taken on admission to the hospital; if there was an abnormal reading, such as an elevated blood glucose level, actions were taken to investigate whether patients had the pre-existing chronic disease prior to or during their stay in the hospital. Furthermore, during the survey, patients also self-reported information about any known chronic disease. For instance, one participant reported a history of hypertension following a previous hypertension diagnosis in this case, a medical certificate or additional medical diagnosis was not required for our study. This is a method widely used to assess MCCs [32]. The number of complications acquired post-stroke onset and the number of pre-exciting MCCs were classified as categories: 0, 1, and ≥ 2.

### Data collection

Data were collected through face-to-face interviews by physiotherapists, occupational therapists, and nurses who were trained for this study. The data were recorded through electronic questionnaires and stored using the online MikeCRM data-management system. Without patient or study team permission, deletion, modification, and sharing of data were prohibited. During the data collection process, the database was supervised by the data administrator. When potentially incorrect or unreasonable data records were found, the data administrator checked with the assessor and corrected the error based on the actual situation.

### Statistical analysis

All analyses were performed using SPSS 23.0 (IBM Corp., Armonk, NY, USA). The number and the frequency (%) distribution of functional state independent variables was reported, and the frequency difference between different functional status groups was tested by the chi-square test. We used the ordinal logistic regression model to carry out the multivariate analysis, comprising patients’ age, sex, BMI, smoking, alcohol consumption, complications, and multiple chronic conditions as covariates. First, we used simple linear regression to detect whether there was multicollinearity between independent variables. If the tolerance is less than 0.1 or the variance inflation factor (VIF) is greater than 10, it indicates collinearity. Second, we used ordinal logistic regression models to explore the factors associated with patients’ poor functional status. Adjusted odds ratio (OR) and 95% confidence intervals (95% CI) of the variables in the final model were reported. A two-tailed *P*-value < 0.05 was considered statistically significant.

## Results

### Demographic, lifestyle characteristics, complications, and multiple chronic diseases of stroke survivors

The average age of the stroke inpatients was 64.38 years old (bedridden group: 66.90 ± 15.28; domestic group: 60.93 ± 15.34; community group: 47.05 ± 13.96). The ratio of males to females was approximately 2:1. The majority of the participants had hypertension (74.3%), and the rates of other variables were: cardiovascular disease (18.1%), diabetes (29.5%), hyperlipidaemia (9.3%), hyperuricemia (3.6%), chronic obstructive pulmonary disease (2.2%), chronic renal insufficiency (7.1%), abnormal liver function (4.2%), chronic bronchitis (2.9%), smoking (8.0%), alcohol use (6.1%), pulmonary infection (35.3%), urinary tract infection (9.2%) and DVT (9.8%) (Table 1). Furthermore, more than half (55.6%) had two or more chronic comorbidities (Table 1).

**Table 1:** Demographic, lifestyle, complications and MCC in different functional status

Characteristics	Total		Functional status						${x}^{2}$	*P*-value
	n	%	Bedridden group		Domestic group		Community group			
			n	%	n	%	n	%		
Type of stroke									5.77	0.056
Ischemic	321	49.69	219	68.22	82	25.55	20	6.23		
Hemorrhagic	325	50.31	247	76.00	58	17.85	20	6.15		
Sex									12.09	**0.002**
Male	443	68.58	308	69.53	98	22.12	37	8.35		
Female	203	31.42	158	77.83	42	20.69	3	1.48		
Age (year)									38.42	** < 0.001**
< 60	258	39.94	156	60.47	71	27.52	31	12.02		
≥ 60	388	60.06	310	79.90	69	17.78	9	2.32		
Duration (month)										
< 12	216	33.43	142	65.74	56	25.93	18	8.33	6.95	**0.031**
≥ 12	430	66.56	324	75.35	84	19.53	22	5.12		
BMI (kg/m^2^)									21.94	**0.005**
≤ 18.4	65	10.42	55	84.62	9	13.85	1	1.54		
18.5–23.9	395	63.30	294	74.43	78	19.75	23	5.82		
24–27.9	143	22.92	88	61.54	44	30.77	11	7.69		
≥ 28	21	3.36	10	47.62	8	38.01	3	14.29		
Smoking									0.86	0.650
Yes	50	7.99	39	78.00	9	18.00	2	4.00		
No	576	92.01	415	72.05	127	22.05	34	5.90		
Alcohol consumption									4.66	0.097
Yes	38	6.06	33	86.84	3	7.89	2	5.26		
No	589	93.94	422	71.65	132	22.41	35	5.94		
Pulmonary infection									79.67	** < 0.001**
Yes	228	35.29	213	93.42	13	5.70	2	0.88		
No	418	64.71	253	60.53	127	30.38	38	9.09		
Urinary tract infection									6.94	0.139
Yes	59	9.20	51	86.21	7	12.07	1	1.72		
No	587	90.87	415	70.70	133	22.66	39	6.64		
Deep venous thrombosis									8.21	**0.017**
Yes	63	9.75	55	87.30	7	11.11	1	1.59		
No	583	90.25	411	70.50	133	22.81	39	6.69		
Complications									65.20	** < 0.001**
0	319	49.40	186	58.31	102	31.97	31	9.72		
1	236	36.50	194	82.20	34	14.41	8	3.39		
≥ 2	91	14.10	86	94.51	4	4.40	1	1.10		
Hypertension									3.04	0.219
Yes	480	74.30	339	70.63	112	23.33	29	6.04		
No	166	25.70	127	76.51	28	16.87	11	6.63		
Cardiovascular disease									1.94	0.379
Yes	117	18.11	86	73.50	27	23.08	4	3.42		
No	529	81.89	380	71.83	113	21.36	36	6.81		
Diabetes									4.30	0.116
Yes	190	29.46	142	74.74	42	22.11	6	3.16		
No	455	70.54	323	70.99	98	21.54	34	7.47		
Hyperlipidemia									1.05	0.593
Yes	60	9.29	41	68.33	16	26.67	3	5.00		
No	586	90.71	425	72.53	124	21.16	37	6.31		
Hyperuricemia									1.97	0.374
Yes	23	3.56	15	65.22	5	21.74	3	13.04		
No	623	96.44	451	72.39	135	21.67	37	5.94		
Chronic obstructive pulmonary disease									2.99	0.224
Yes	14	2.17	11	78.57	1	7.14	2	14.29		
No	632	97.83	455	71.99	139	21.99	38	6.01		
Chronic renal insufficiency									0.54	0.765
Yes	46	7.12	35	76.09	8	17.39	3	6.52		
No	600	92.88	431	71.83	132	22.00	37	6.17		
Abnormal liver function									3.38	0.185
Yes	27	4.18	23	85.19	2	7.41	2	7.41		
No	619	95.82	443	71.57	138	22.29	38	6.14		
Chronic bronchitis									3.15	0.207
Yes	19	2.94	17	89.50	2	10.50	0	0.00		
No	627	97.06	449	71.60	138	22.00	40	6.40		
Multiple chronic conditions									8.00	0.104
0	72	11.10	55	76.39	11	15.28	6	8.33		
1	215	33.30	142	66.05	57	26.51	16	7.44		
≥ 2	359	55.60	269	74.93	72	20.06	18	5.01		

Significant *p*-values are bolded

### Functional status

The overall prevalence of the bedridden group was 72.14%, the prevalence of the domestic group was 21.67%, and that of the community group was 6.19% (Table 2). Table 2 reports responses of stroke survivors to each item of the LS scale regarding functional status. In the bedridden group, participants reported inability to eat most frequently (73.39%), followed by bowel and bladder management (69.74%) and entertainment (69.53%), and 74.29% of the domestic group participants had a severe problem performing housework (Table 2).

**Table 2:** Stroke survivor's responses to each item on the LS scale of functional status

LS group (n, %)	Item	1point		2 points		3 points	
		n	%	n	%	n	%
Bedridden group (466, 72.14%)	Bowel and bladder management	325	69.74	95	20.39	46	9.87
	Feeding	342	73.39	94	20.17	30	6.44
	Entertainment	324	69.53	127	27.25	15	3.22
Domestic group (140, 21.67%)	Toileting	18	12.86	86	61.43	36	25.71
	Bathing	68	48.57	54	38.57	18	12.86
	Housework	104	74.29	34	24.29	2	1.43
Community group (40, 6.19%)	Community mobility	5	12.50	11	27.50	24	60.00
	Shopping	3	7.50	17	42.50	20	50.00
	Social interaction	1	2.50	13	32.50	26	65.00

### Independent variables related to poor functional status

The complete model includes all variables listed in Table 1, except for the number of MCC and complications. Therefore, the model is adjusted for all the variables in Table 3. The VIF of the variables included in the model is less than 3.0. In the final model, sex, age, stroke duration, pulmonary infection and DVT were significantly correlated with poor functional status (Table 3). The risk of poor function (OR = 2.43, 95% CI: 1.56 – 3.80) in the elderly group (≥ 60 years old) was more than twice that in the young group (< 60 years old). Those with a disease duration of more than 12 months had close to twice the risk of poor functional status (OR = 1.72, 95% CI: 0.97–3.07) than those whose disease duration was within 12 months. The risk was also close to or greater than double among females than males (OR = 1.73, 95% CI: 1.08–2.77), and among those with pulmonary infection (OR = 11.58, 95% CI: 6.02–22.27) and DVT (OR = 3.05, 95% CI: 1.24–7.53).

**Table 3:** Factors associated with poor functional status

Characteristics	Adjusted odds ratio^a^	95% Confidence Interval		P
		Lower	Upper	
Sex				
Male	Ref			
Female	**1.73**	**1.08**	**2.77**	**0.022**
Age(year)				
< 60	Ref			
≥ 60	**2.39**	**1.55**	**3.69**	** < 0.001**
Duration of stroke(month)				
< 12	Ref			
≥ 12	**1.94**	**1.28**	**2.95**	**0.002**
Alcohol consumption				
No	Ref			
Yes	3.03	0.92	10.01	0.068
Pulmonary infection				
No	Ref			
Yes	**10.91**	**5.81**	**20.5**	** < 0.001**
Deep venous thrombosis				
No	Ref			
Yes	**3**	**1.28**	**7.04**	**0.012**

^a^ Adjusted for all the factors shown in Table 1; Significant *p*-values are bolded

## Discussion

This study aimed to assess the correlates of functional status based on the LS scale among patients with stroke in hospital settings in Shenzhen, China. We found that 74.12% of stroke survivors, categorized as the bedridden group had poor functional status, 21.67% of patients, the domestic group, had moderate functional limitation, and 6.19%, the community group, had mild functional restriction. This observational study showed that older age, sex, duration of a stroke, pulmonary infection and DVT were associated with poor functional status among inpatients with stroke.

In our study, compared with individuals aged < 60 years, the poor functional status of stroke survivors was 2.43 times higher among individuals aged ≥ 60 years. This finding is similar to other studies regarding stroke functional outcomes, showing that the risk of stroke and poor outcomes were significantly associated with age [10, 33, 34]. Once stroke occurs, the regenerative potential decreases, and the inflammatory responses to this disease increase in the elderly [35, 36]. Furthermore, we found that the proportion of stroke survivors aged < 60 years in our study was greater than that in a study conducted in Singapore [24]. This may be because Shenzhen is one of the most developed cities in China. It attracts numerous young people from different parts of China and globally to seek career opportunities. The 2020 census reported that the proportion of permanent residents aged ≥ 60 years in Shenzhen was as low as 5.36% [37]. The other possible reason for the trend of stroke in younger people in recent years is the increased stress and intake of refined cereal products and salt in China [18].

We also noted that 75.3% of patients with a duration of disease greater than one year had poor functional status, while 65.7% of stroke survivors with a duration of disease less than one year had poor functional status. Among hospitalized stroke survivors, the functional status of patients with a duration of disease greater than one year also appeared to be worse than those with a duration of less than one year (Table 1 and Table 3). This is not surprising because stroke survivors with good functional status were often discharged home within 1 year of stroke onset. In contrast, patients with poor functional ability were transferred from one hospital to another to receive continuous medical and rehabilitation therapies. Patients were unwilling to be discharged home and receive such therapies in the community because the quality of primary care in China is still not ideal [38]. People prefer to be hospitalised if they are financially capable and are willing to receive higher-level care treatments for a long period [39]. For this reason, stroke survivors hospitalized > 12 months duration have poorer functional status in our study.

Hospitals in China are classified into three levels, primary, secondary and tertiary, according to several factors such as the scale of hospitals, the number of beds, medical equipment and facilities, and the specialties of the healthcare professionals [39, 40]. Tertiary hospitals have the most medical resources and are generally equipped with better-qualified healthcare professionals. More tertiary hospitals are found in urban cities than economically less developed cities due to huge socio-economic gaps and geographical inequalities in the distribution and utilization of medical resources, especially between urban and rural areas, and among different levels of hospitals [40]. Because of the poorly developed hierarchical medical system in China [39], medical resources and funding are mostly concentrated in secondary and tertiary hospitals, where there are excellent rehabilitation facilities and professional therapists for stroke survivors. Patients can be referred to multiple hospitals repeatedly, regardless of disease severity, and are more willing to receive treatments in secondary and tertiary hospitals compared with community rehabilitation practice or primary level hospitals due to a lack of a strict referral and check-in system in the public healthcare system [38]. This allows patients to visit different hospitals and have a long hospital stay.

The present study also indicated that a sex difference in functional status exists among stroke survivors. We observed that women were more likely to have a severe stroke and were more likely to be assessed as the bedridden group than men. Women in our study had more than double the odds of poor functioning (Table 3). This is similar to several studies, which show that women have higher 1‐month case fatality and lower 1-year survival after stroke [7, 8, 41]. One plausible explanation is that the age of the women was older than that of the men in our study (Supplementary Table 1). Previous studies also found that compared with males, female stroke survivors have more severe strokes, less aspirin administration and are likely to receive lower quality of care [42]. Some risk factors may also contribute to this situation, such as atrial fibrillation and hypertension which are more common in women [43, 44].

Unlike many previous studies that describe alcohol consumption as a complicated epidemiological risk factor of stroke [11, 33], it was not identified as a risk factor in our study in the final ordinal logistic model (Table 3). Yet, the proportion (86.84%) of stroke survivors with poor functional status among those who consume alcohol was higher than in those who did not(71.65%) (Table 1). These results suggested that reducing alcohol consumption might be associated with ADL dependence of stroke survivors. High-dose alcohol consumption is associated with a high stroke incidence rate and stroke mortality, but low-dose (i.e., < 30 to 40 g/day, 1 to 2 standard drinks/day) alcohol consumption may have a protective effect on stroke [11, 33]. Future research should focus on how the quantity of alcohol consumed affects the levels of functional ability after stroke onset.

Being underweight is highly associated with poor functional status after stroke. In the univariate analysis, 84.62% of participants had poor functional status in the underweight group, compared to 61.54% in the overweight group and 47.62% in the obese group (Table 1). The results are similar to previous studies, which reported that overweight/obese stroke patients had better functional outcome and lower mortality than patients with normal/low BMI [45, 46]. People with normal BMI or obese individuals have better nutritional status after vascular events which can significantly facilitate and accelerate recovery [47]. However, the ordinal logistic analysis model revealed that BMI is not a risk factor for poor functional ability in our study. A plausible explanation for this is that there is not a sufficient sample size in the obese group (only 21 people were classified as obese). Future research should explore the association between different BMI and levels of functional ability in stroke survivors.

Previous studies have found that post-stroke infections can worsen stroke prognosis [48, 49]. Pulmonary infection is considered the most common infection after stroke and is associated with relatively higher mortality risk [48]. In our study, the overall infection rate of pulmonary infection was 35.3%, and urinary tract infections occurred in 9.2% of patients. Pulmonary infection was associated with poor functional status with an OR of 10.91, similar to a previous study in which patients with a higher stroke severity had higher infection rates, especially for the pulmonary infection [14]. This is because infections could lead to immobilization, general frailty and rehabilitation delay which affect stroke survivors' functional outcomes [13, 49]. Pulmonary infections can also contribute to other complications such as DVT [12].

In our study, patients with DVT had poorer functional status (87.30%) than those without (70.50%) (Table1). Some commonly studied factors related to DVT such as immobilization, older age, and pulmonary infection are associated with poor functional ability of stroke survivors [12]. Patients are more likely to have a stroke in the first year after having DVT [50]. In the present study, we explored the relationship between DVT and stroke in terms of functional ability and found that having DVT was associated with tripled odds of a poorer level of functional ability (Table3). However, since this study was a cross-sectional study, it was impossible to determine the causal relationship between these risk factors and poor functional status; thererore only the correlation between these factors and poor functional status was determined.

Unlike other studies, MCC was not a risk factor for poor functional status in the logistic analysis in this study. Previous studies have shown that MCC affects post-stroke neuropsychological adaptation, and stroke survivors with MCC have weak physical and/or cognitive abilities, worse hemodynamics, and worse collateralization of flow after arterial occlusions, which hinder functional recovery after stroke [19, 51–55]. A potential explanation for this difference is that almost 90% of the patients had more than one chronic condition(Table 1); there was no difference in the proportion of MCC among stroke survivors with different levels of functional ability in our study, and the proportion of patients without MCC was too small to influence the statistical analysis results.

The strength of this study is that we comprehensively analyzed factors related to functional status as a whole in hospitalized stroke survivors. Our results can enable physicians to perform early patient management to improve their functional status, such as paying more attention to preventing pulmonary infection and DVT among inpatients with stroke in rehabilitation settings.

Our study has several limitations. As mentioned previously, this study could not determine causal relationships due to the cross-sectional design. Additionally, the hospital sampling method was non-random as the included hospitals were collaborative organizations of our department in Shenzhen, China. The findings are representative of hospitals in Shenzhen, yet may not represent hospitals in other areas (e.g., rural areas) or be generalizable to other settings (e.g., non-hospital environment). Moreover, this study was conducted in secondary and tertiary hospitals where most participants were classified in the bedridden group which may have affected the statistical comparison of relevant data. Further studies should be conducted focusing on stroke survivors in primary care where more stroke survivors with better functional ability can be included.

## Conclusion

In this study, we surveyed stroke survivors’ functional status and explored relevant factors associated with functional status in rehabilitation departments of hospitals in Shenzhen, China. Several factors such as older age, female sex, course of disease longer than 12 months, pulmonary infection and DVT put stroke survivors at nearly two times higher risk of a poorer rehabilitation outcome than others. Therefore we suggest that patients and clinicians should pay close attention to these factors, and measures should be taken to prevent important complications such as pulmonary infections and DVT in stroke survivors. Since all data were obtained in metropolitan areas where the economy is well developed, future studies should be carried out in rural and economically less developed cities. Community practice should also be considered, allowing diverse population sampling to comprehensively analyze risk factors related to the functional status among stroke survivors. This may guide complication prevention strategies and clinical and rehabilitation interventions to improve the functional status of stroke survivors.

## Supplementary Information

**Additional file 1: Table1.** Demographic, lifestyle, complications and MCC by the gender (*n* =646).

## Data Availability

All the summarized and analyzed data during this study are included in this published article; the original data in this study are available from the corresponding author upon reasonable request.

## Abbreviations

MCC: Multiple Chronic Condition
ADL: Activities of Daily Living
BMI: Body mass index
DVT: Deep Vein Thrombosis
LS: Longshi Scale
VIF: Variance Inflation factor
OR: Odds Ratio
CI: Confidence Interval
