# Supplementary material for: Functional status and its related factors among stroke survivors in rehabilitation departments of hospitals in Shenzhen, China: a cross-sectional study
Source: BMC Neurol. 2022 May 11;22:173. doi: 10.1186/s12883-022-02696-0 (PMC9092870; doi:10.1186/s12883-022-02696-0)
Supplement: Supplementary file 1 — Additional file 1: Table1. Demographic, lifestyle, complications and MCC by the gender (n =646). [file 12883_2022_2696_MOESM1_ESM.docx]

# Supplementary table

Table1 Demographic, lifestyle, complications and MCC by the gender (n=646)

| Characteristics | Total | | Gender | | | | $x^{2}$ | P-value |
| --- | --- | --- | --- | --- | --- | --- | --- | --- |
|  | n | % | Female  (n=203，31.4%) | | Male  (n=443,68.6%) | |  |  |
|  |  |  | n | % | n | % |  |  |
| Type of stroke |  |  |  |  |  |  | 1.9 | 0.168 |
| Ischemic | 321 | 49.69 | 109 | 53.69 | 212 | 47.86 |  |  |
| Hemorrhagic | 325 | 50.31 | 94 | 46.31 | 231 | 52.14 |  |  |
| Age (year) |  |  |  |  |  |  | **25.31** | **<0.001** |
| ＜60 | 258 | 39.94 | 52 | 25.62 | 206 | 46.50 |  |  |
| ≥60 | 388 | 60.06 | 151 | 74.38 | 237 | 53.50 |  |  |
| Duration(month) | |  |  |  |  |  | 0.27 | 0.605 |
| ＜12 | 216 | 33.44 | 65 | 32.02 | 151 | 34.09 |  |  |
| ≥12 | 430 | 66.56 | 138 | 67.98 | 292 | 65.91 |  |  |
| BMI (kg/m2) |  |  |  |  |  |  | 9.26 | **0.055** |
| ≤18.4 | 65 | 10.42 | 28 | 14.21 | 37 | 8.67 |  |  |
| 18.5-23.9 | 395 | 63.30 | 112 | 56.85 | 283 | 66.28 |  |  |
| 24-27.9 | 143 | 22.92 | 48 | 24.37 | 95 | 22.25 |  |  |
| 28-32 | 19 | 3.04 | 9 | 4.57 | 10 | 2.34 |  |  |
| ＞32 | 2 | 0.32 | 0 | 0 | 2 | 0.47 |  |  |
| Smoking |  |  |  |  |  |  | **22.06** | **<0.001** |
| Yes | 50 | 7.99 | 1 | 0.51 | 49 | 11.45 |  |  |
| No | 576 | 92.01 | 197 | 99.49 | 379 | 88.55 |  |  |
| Alcohol consumption | |  |  |  |  |  | **15.56** | **<0.001** |
| Yes | 38 | 6.06 | 1 | 0.51 | 37 | 8.60 |  |  |
| No | 589 | 93.94 | 196 | 99.49 | 393 | 91.40 |  |  |
| Pulmonary infection | |  |  |  |  |  | 0.22 | 0.639 |
| Yes | 228 | 35.29 | 69 | 33.99 | 159 | 35.89 |  |  |
| No | 418 | 64.71 | 134 | 66.01 | 284 | 64.11 |  |  |
| Urinary tract infection | |  |  |  |  |  | **3.61** | **0.057** |
| Yes | 59 | 9.13 | 25 | 12.32 | 34 | 7.67 |  |  |
| No | 587 | 90.87 | 178 | 87.68 | 409 | 92.33 |  |  |
| Deep venous thrombosis | | |  |  |  |  | **2.74** | **0.098** |
| Yes | 63 | 9.75 | 14 | 6.90 | 49 | 11.06 |  |  |
| No | 583 | 90.25 | 189 | 93.10 | 394 | 88.94 |  |  |
| Complications |  |  |  |  |  |  | 3.35 | 0.188 |
| 0 | 319 | 49.38 | 104 | 51.23 | 215 | 48.53 |  |  |
| 1 | 236 | 36.53 | 65 | 32.02 | 171 | 38.60 |  |  |
| ≥2 | 91 | 14.09 | 34 | 16.75 | 57 | 12.87 |  |  |
| Hypertension |  |  |  |  |  |  | 0.55 | 0.457 |
| Yes | 480 | 74.30 | 147 | 72.41 | 333 | 75.17 |  |  |
| No | 166 | 25.70 | 56 | 27.59 | 110 | 24.83 |  |  |
| Cardiovascular disease | |  |  |  |  |  | **5.07** | **0.024** |
| Yes | 117 | 18.11 | 47 | 23.15 | 70 | 15.80 |  |  |
| No | 529 | 81.89 | 156 | 76.85 | 373 | 84.20 |  |  |
| Diabetes |  |  |  |  |  |  | 0.61 | 0.435 |
| Yes | 190 | 29.46 | 64 | 31.53 | 126 | 28.51 |  |  |
| No | 455 | 70.54 | 139 | 68.47 | 316 | 71.49 |  |  |
| Hyperlipemia |  |  |  |  |  |  | 0.29 | 0.588 |
| Yes | 60 | 9.29 | 17 | 8.37 | 43 | 9.71 |  |  |
| No | 586 | 90.71 | 186 | 91.63 | 400 | 90.29 |  |  |
| Hyperuricemia | |  |  |  |  |  | 0.01 | 0.917 |
| Yes | 23 | 3.56 | 7 | 3.45 | 16 | 3.61 |  |  |
| No | 623 | 96.44 | 196 | 96.55 | 427 | 96.39 |  |  |
| Chronic obstructive pulmonary disease | | | |  |  |  | 3.92 | **0.048** |
| Yes | 14 | 2.17 | 1 | 0.49 | 13 | 2.93 |  |  |
| No | 632 | 97.83 | 202 | 99.51 | 430 | 97.07 |  |  |
| Chronic renal insufficiency | | |  |  |  |  | 1.3 | 0.255 |
| Yes | 46 | 7.12 | 11 | 5.42 | 35 | 7.90 |  |  |
| No | 600 | 92.88 | 192 | 94.58 | 408 | 92.10 |  |  |
| Abnormal liver function | |  |  |  |  |  | 0.4 | 0.53 |
| Yes | 27 | 4.18 | 7 | 3.45 | 20 | 4.51 |  |  |
| No | 619 | 95.82 | 196 | 96.55 | 423 | 95.49 |  |  |
| Chronic bronchitis | |  |  |  |  |  | 0.27 | 0.606 |
| Yes | 19 | 2.94 | 7 | 3.45 | 12 | 63.16 |  |  |
| No | 627 | 97.06 | 196 | 96.55 | 431 | 68.74 |  |  |
| Multiple chronic conditions | | |  |  |  |  | 1.51 | 0.468 |
| 0 | 72 | 11.15 | 25 | 12.32 | 47 | 10.61 |  |  |
| 1 | 215 | 33.28 | 61 | 30.05 | 154 | 34.76 |  |  |
| ≥2 | 359 | 55.57 | 117 | 57.64 | 242 | 54.63 |  |  |

Significant p-values are bolded.
